# Shifting temporal patterns in physical usage of a health sciences library following the extension of operational hours

**DOI:** 10.5195/jmla.2024.1812

**Published:** 2024-10-01

**Authors:** G.J. Corey Harmon, Kerry Sewell

**Affiliations:** 1 harmong18@ecu.edu, Head of Access Services and Library Assistant Professor, Laupus Health Sciences Library, East Carolina University, Greenville, NC; 2 browderk@ecu.edu, Research Development Director, College of Health & Human Performance, East Carolina University, Greenville, NC

**Keywords:** Physical Library Usage, Extended Hours, Weekend Library Hours, Health Sciences Library Hours

## Abstract

**Background:**

Students regularly state a strong preference for increased library operational hours to accommodate learning needs. While many academic libraries provide extended hours, academic health sciences libraries appear to adopt such models less frequently. This is not due to lower levels of student demand for extended hours.

**Case Presentation:**

In response to student preferences for increased hours, our institution shifted its budget to provide an additional 10 weekend hours (Friday-Sunday). This study is a post-hoc assessment of temporal changes in physical library use over weekend days pre- and post-implementation of extended hours. This study used (non-exam) weekend physical library usage data one year before and after implementing extended hours (January 2018–January 2020). We ran descriptive statistics to assess the hourly use pattern changes in physical library usage.

**Conclusion:**

For the regular academic year, students arrived earlier and stayed later, with less severe hourly peaks in opening and closing times physical use patterns seen in the pre-implementation period. Overall, we saw increases in all three areas of physical usage data studied. The data provides evidence of the true need for extended hours among users, along with hourly patterns reflecting a better match of hours with patron study patterns.

## BACKGROUND

Anecdotal evidence, along with published literature, makes clear the near-universal nature of student preferences for extended operational hours to accommodate learning needs, with library directors noting that providing study space is their top priority [1–8]. Students usually request unlimited, 24-hour access [9] and academic library administrators have listened. Since the mid-1990s, the number of Association of Research Libraries (ARL) libraries reporting use of one of the various 24-hour operating models increased from 5.15% [10] to 91% [1]. Concomitantly, the number of publications on the practice and effect of extending operating hours in academic libraries has risen [1–5,10–15].

The same is not true of medical and health sciences libraries (HSLs), which are not exempt from student demand for extended hours [11,16–17]. The last broad study of HSLs’ operational hours was published in 2003, reporting that the number of service hours among Association of Academic Health Sciences Libraries (AAHSL) member libraries increased by only 5% or 4 hours per week between 1978-2002 [18]. More recent AAHSL member operational hours data are not freely available. Beyond the outdated, available AAHSL data and a smattering of articles, published evidence about HSLs’ operating hours is largely presented through posters at regional library conferences [19,20]. While HSLs may be included in broader surveys of libraries, they face unique challenges with budgets and user populations that differ from their Academic Library counterparts [21,22]. HSLs should be considered separately to better understand their operation and usage.

The reasons for the relative paucity of literature on this topic from HSLs are unknown. One publication on extending hours in a HSL indicates that budgetary reasons drove decisions about extended hours, noting that user demands for extended weekend hours could only be met by cutting weeknight hours [12]. Budgetary considerations for extended hours are more frequently discussed in academic library publications, which describe budgetary issues such as extra staffing, security, and other library services [1,4,5,10,13,23]. For HSLs, which may have smaller budgets than their Academic Library counterparts [24], these considerations might weigh more heavily in operational hours decisions, which must be balanced against increasing, competing demands [22,25].

When library budgets can accommodate changes to operational hours, the costs must be weighed against expected levels of library use during extended hours. Projecting levels of use of extended hours may involve examination of entry and exit gate counts around thencurrent opening and closing times, service statistics, interviews, or survey data on the typical times of day that students study [23]. These pre-implementation data points are critical and may lead libraries to reduce their hours of operation rather than extending them [26].

Following the extension of operational hours, post-hoc assessments are equally important, providing evidence for (or against) continuation of a model. Post-hoc assessments may involve operational data, observational data (such as head counts), time use studies, or interview data elucidating students’ reasons for using the library during extended hours. Published, post-hoc studies reveal that the primary reason students use the library during extended hours is as a study space [14,27–29], with reference and operational services going largely unused during extended hours [1,14,15]. Many libraries subsequently reduce or eliminate such services. Libraries that do offer extended hours services typically focus on circulation [4,15,27]. As previously noted, most of these statistics come from academic libraries, but the findings are mirrored in two HSL-based studies, which found that most survey respondents neither borrowed materials, requested articles, nor requested skilled assistance from staff during extended hours [30,31].

Both survey data from users about the adequacy of library hours and post-hoc data on library usage are critically important, allowing libraries to make informed decisions in the face of competing budgetary pressures and user demands. This study describes the process of implementing and analyzing the impact of extended library hours in an academic HSL through the lens of physical usage of the library’s spaces and resources. This was accomplished by examining the library’s gate count, circulation, and study room reservations both pre- and post-extended hours.

## CASE PRESENTATION

The Laupus Health Sciences Library is a large, academic medical library situated on East Carolina University (ECU) Health Sciences Campus in a small metropolitan area. Laupus Library serves [>4,500] students enrolled in four colleges and schools on that campus (including the School of Medicine, the School of Dental Medicine, the College of Allied Health Sciences, and the College of Nursing), along with the university’s teaching hospital, ECU Health Medical Center. Laupus Library also indirectly supports the health-adjacent disciplines and classes on the academic campus. Most of the health sciences programs are weekday, in-person programs, with few select distance education programs.

Prior to 2019, Laupus Library was open 95.5 hours per week (7:30 am–12:00 am Monday through Thursday, 7:30 am–5:00 pm Friday, 9:00am-5:00pm on Saturday, and 12:00 pm–12:00 am on Sunday). The library contains 28 study rooms that students could reserve for 3 hour blocks up to two times per day. Over the weekend, Saturday service included both a reference librarian and a full-time paraprofessional staffing the sole point of service, between 9:00 am–5:00 pm. Sundays were exclusively staffed by paraprofessionals and students. This model was used for at least a decade of library operations, during which time students regularly requested extended hours via direct emails and satisfaction surveys.

In 2018, the leadership of the departments primarily responsible for providing circulation, reference, technical, and operational support changed. The two new leaders oversaw the User Services (US) department, consisting of reference and instruction librarians and a User Engagement paraprofessional, as well its subdepartment, Access Services (AS), managed by a librarian and including paraprofessionals responsible for staffing the service desk. After reviewing a decade’s worth of reference statistics and student requests for extended hours, the new leaders reassessed service desk models, including service desk staffing and extended operational hours.

Changes in operational hours required a reconsideration of staffing to accommodate the extension of hours. In lieu of moving existing paraprofessional and librarian schedules, US and AS leadership proposed a new part-time (.5 FTE) AS position to cover the extended hours. The position was viewed as critical to ensuring that the part-time paraprofessional would be available on Saturdays to assist librarians with any unusual operational questions. Simultaneously, the number of full-time personnel needed to staff the service desk on Saturdays was reassessed. The proposed staffing model included either a librarian or a full-time paraprofessional staffing the service desk, as well as the part-time position, rather than two full-time employees. These combined changes were meant to accommodate student learning needs and better utilize the time of those providing weekend user-facing services.

In considering weekend extended hours, six schedule options were originally offered to library administration. The final expanded operational model chosen by administration was a mix of several of the models, increasing operations by 10 hours over the weekend. The model was chosen due to its benefits for the personnel working weekends (including student workers) and for the library’s budget. Planned absences were covered by full-time staff shifting their hours and unplanned absences were managed by student workers and voluntary support from other employees. Three hours each were added on Friday and Saturday (5:00 pm–8:00 pm) and four hours were added on Sunday (8:00 am–12:00 pm), increasing the library’s operating hours to 105.5. The .5 FTE staff position was approved to cover extended hours between Friday and Sunday, funded through the personnel budget. Library administration reported shifting its budget to accommodate the changes rather than requiring new funds for the .5 FTE position, notably through an attritiondriven reduction in the number of student workers. The new position, along with the change to desk staffing, reduced the number of Saturdays that other members of US had to work from monthly for paraprofessionals and bimonthly for librarians to twice per semester for each respective employee type.

Although the library opened a virtual reality lab during this time, it was not open during the extended hours. No other programmatic or advertising changes were made beyond announcing the extended operational hours via physical signage, the health campus listserv, and liaison outreach to their colleges/schools. This announcement was sent just prior to the change and upon its initial implementation.

## METHODS

This study retrospectively assessed the effect of extended weekend hours on physical use of the library using gate counts, circulation statistics, and room reservations between January 14, 2018, and January 12, 2020. The institution’s University and Medical Center IRB confirmed that because this study used standard operational data to assess how aggregate weekend academic library use changed after operational changes at an institution, involving no hypothesis and not collecting information about the library’s individual users in response to the change, it did not meet the federal definition of Research and no Human Subjects Research considerations or regulations were triggered by the reported assessment. Additionally, gate count and circulation data contained no identifiable information. Only the raw room reservation data included identifiable information and all user identifiers were anonymized and removed.

The dates chosen represent one year each prior to and following the implementation of extended hours. Although extended hours continued until the onset of the pandemic, this choice of time periods allows for a matched sample, pre- and post-implementation, aligning with the starts of spring semesters.

The three metrics chosen — gate counts, circulation, and room reservations — provide a rich picture of the physical use of the library during operating hours, covering building occupancy levels throughout the day, use of materials in the library’s physical collections, and use of dedicated study spaces. The library gate count data were taken from Sensource hardware and software used in the library to capture entries and exits, along with their time stamps [32]. Circulation data were harvested from the Integrated Library System (ILS), SirsiDynix Symphony. Room reservations were captured through an internally built room reservation system.

All data from these systems were exported as either .xlsx or .csv files and subsequently cleaned and prepared for use by adding additional data points such as whether the date and time for a given case occurred during the regular academic semester, during extended hours, or during an exam period. Gate entries occurring during time periods when the library was closed to all students, such as state and federal holidays, were removed from the dataset. The cases that occurred during exam periods were removed from analysis files because exam periods vastly alter student library use and study behaviors and because, even prior to the implementation of extended hours, exam periods already included short-term extended operating hours.

Descriptive statistics were used to assess hourly use of physical spaces and resources, pre- and post-extended hours for the following data points: total number of users per weekend day by hour (assessed by gate counts), total checkouts per weekend day by item type and by hour, and total number of room reservations and room reservation hours by weekend day. Hourly usage trends were generated as multiple line graphs, paneled by weekend day and semester. Data analysis was performed in SPSS. Cleaned and de-identified data sets are provided in Open Science Framework.

## RESULTS

Laupus Library’s statistics showed altered hourly physical use patterns across all three of the data sets that we collected, though there was variation by weekend day and by semester for hourly patterns in gate counts, room reservations, and circulation.

### Gate Count

**Figure 1:**
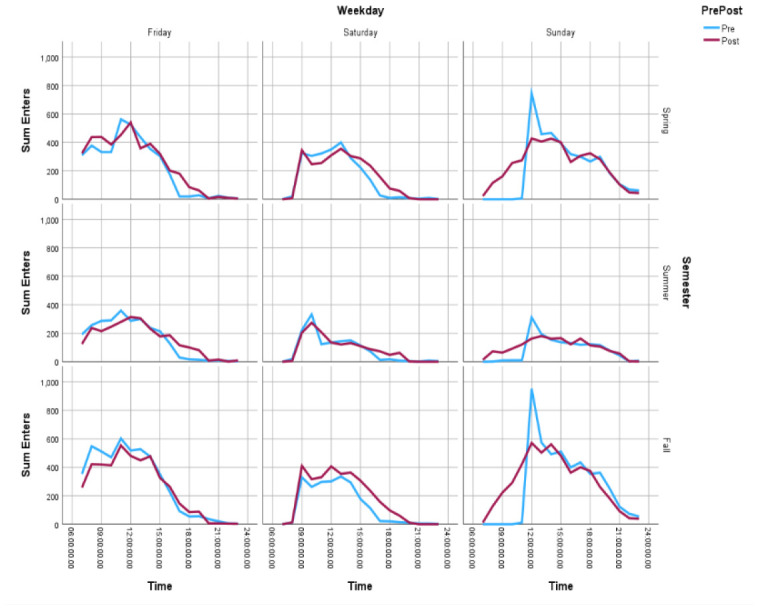
Sum of Entries by Time of Day for Friday, Saturday, and Sunday, by Semester

**Figure 2:**
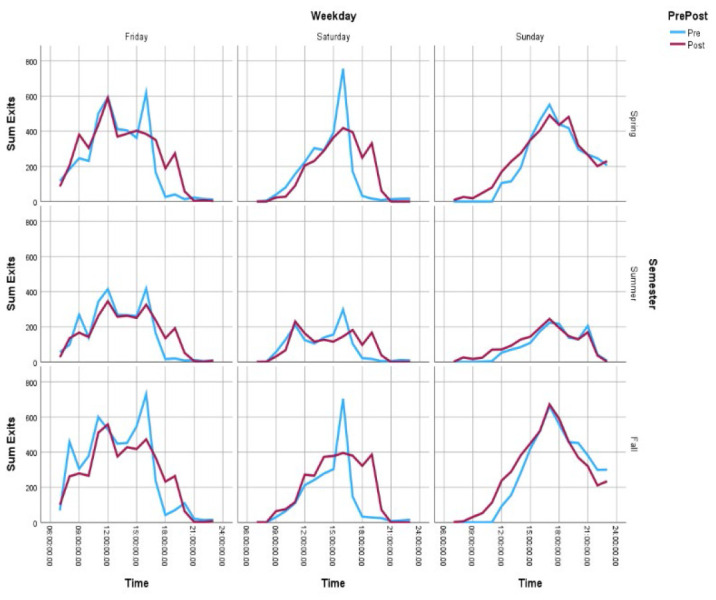
Sum of Exits by Time of Day for Friday, Saturday, and Sunday, by Semester

Gate counts of entries were higher post-implementation, across all three weekend days. Gate count totals were consistently highest on Fridays, lowest on Saturdays, and rising again on Sundays, both pre- and post-implementation. Hourly gate entry patterns reveal changing use: while gate entries were similar on Fridays and Saturdays, Sunday gate count entries show an altered pattern, with a gentle slope at the start of the day, as opposed to the large spike in entries at opening, pre-implementation. This changed pattern held true across semesters. Exit counts by weekend day across semesters also show that extended hours led to a significantly reduced spike at closing time on Fridays and Saturdays. Sunday exit data is mirrored across pre- and post-implementation. Gate count entry data also indicate that the proportion of entries during the extended hours as a proportion of the total entries for a given weekend day varied by weekend day (Friday: 8%; Saturday: 11%; Sunday: 21%). The data are available in Supplementary Table 1. Figures 1 and 2 show the gate count entries and exits, respectively, by time of day for each weekend day, pre- and post-implementation of extended hours.

### Room Reservations

The total hours of room reservations occurring during extended hours in 2019 accounted for 17.73%, 17.34%, and 15.91% of all reservations during the Spring, Fall, and Summer Semesters respectively (Supplementary Table 2). Supplementary figures 1–3 show the changes in hourly patterns of reserved study room use as well as start and end times pre- and post-implementation of extended hours. The authors note that the data indicate that room reservations were higher post-implementation, though not uniformly. Start times on Spring and Fall Semester Sundays, post-implementation, show a gradual increase in room reservation start times throughout the morning, as opposed to the steeper slope of study room reservation hours starting at noon, pre-implementation. Likewise, the room reservations on Fridays and Saturdays show much higher hourly rates of reserved study room use in the late afternoon, in addition to use of study rooms during extended hours. General room reservation patterns follow library data trends more broadly, with Sundays having the highest level of use of reservable study rooms. The authors note an incidental finding of a consistent 3 p.m. dip in reserved study room use.

### Circulation

**Figure 3:**
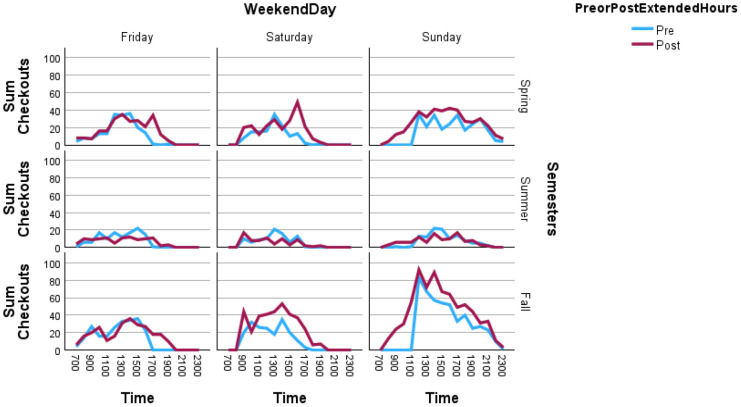
Changes in Circulation by Hour, Pre- and Post-Implementation of Extended Hours, by Weekend Day and Semester

Circulation data increased following implementation of extended hours. Overall, the number of items checked out increased by 40.18% during non-exam periods between the pre- and post-implementation periods of study, though summer circulation decreased. Circulation patterns by time of day changed most notably on Saturdays during the period of study, with higher and shifted peaks during the Saturday afternoons of the Spring and Fall Semesters. Examining circulation by item types, results indicate that the anatomical model collection, which is library-use only, saw a large increase in use during the extended hours, with particularly high usage on Sundays in the Spring and Fall Semesters, even within the hours of pre-implementation operation. Patterns of use by time of day were typically similar, except for Saturdays, when anatomical model use peaked late in the day, indicating high levels of student use during extended evening hours. This increase in usage was also observed in other library-only items like dry-erase marker kits. Items that could be used outside the library (typically books and laptops) saw either declines in checkouts during extended hours or small increases. Data on circulation are presented in Figure 3 and Supplementary Figure 4. The sum of checkouts for these year-long periods as a total are nonetheless low.

## DISCUSSION

The results of the study signal that extending operational hours based upon students’ expressed need resulted in altered temporal patterns of physical space and resource usage, with students entering, using dedicated study spaces, and checking out library-only items during those extended hours. Student entries and room reservations had less marked peaks around openings on Sundays and closings on Fridays and Saturdays. The data also show increased use of resources (spaces and collections) overall, with variation by semester. The latter findings are notable in the face of published data from the profession describing dropping gate counts over the last two decades [33–36] as electronic resources make a substantial portion of library collections accessible anywhere.

The observed temporal changes in physical library usage following implementation of extended hours underline the ongoing value of the library as space beyond the hours when classes occur or during typical weekend ‘business hours.’ This also ties neatly with discussions of libraries as a “third place,” with their own unique environment [37–39] possibly offering advantages over other locations for study for the student accessing the library post-extended hours. The increase in library use for the weekend days and times during which operational hours were extended is particularly noteworthy, given that Friday and Saturday evenings and early Sunday mornings cut into the traditional social weekend when students typically engage in social, employment, and religious activities, or weekend morning sleep [40, 41].

The authors note that not all weekends, across all semesters, saw the same, or similar rates in use of the library during extended hours. This may reflect changing student priorities across the Fall, Spring, and Summer Semesters. These disparities are most notable in the Fall Semester, which the authors suggest has stronger attractions altering study behaviors, such as major American holidays, campus events such as homecoming, and campus social and cultural experiences, such as establishing and rekindling friendships after the summer, Greek organization initiations and events, and big-time sporting events [42].

The results of the study allowed Laupus Library to confirm that student demand for extended hours was an expression of true need, rather than a desire for unfettered access to resources, regardless of actual need or use. Extreme peaks around pre-implementation opening and closing times indicate pent-up demand, reflecting the times when many students would have come earlier or stayed later. Moderated temporal patterns in the post-implementation data indicate that operational hours better fit extant academic study patterns among users.

The data additionally provides some sense of the value of extending hours to Laupus Library’s users. The authors did not attempt to calculate a monetary value of the extended hours, an exercise which would be difficult, and even problematic in the paradigm of education and research being the primary ‘business’ foci of higher education institutions. Indeed, determining a Return On Investment (ROI), as applied in business, can be problematic in any library context, and may not be the most effective means of showing the value of operating hours changes to those assessing the value of library services [43]. Instead, assessing how students, faculty and staff choose to use their time during hours that they might otherwise spend pursuing academic, financial, or psychosocial goals in other locations shows value in compelling ways. As Shepherd, Vardy, & Wilson [44] note, “… time is a limiting factor for everyone, [therefore] how people use their waking hours reflects their values, priorities and personal interests” (p. 450).

Importantly, the change in staffing patterns did not impose a high level of burden on pre-existing positions or the library’s budget. Although not formally assessed, library employees expressed satisfaction with the altered weekend schedule. Additionally, no user complaints about reduced weekend access to librarians were submitted to administrators during or after the study period. This, along with the temporal shifts in physical use, was an important confirmation for library administrators. As previously noted, post-hoc assessments such as this one are key to ensuring that limited budgets are best allocated to balance user needs and responsible accounting.

Laupus Library, like most other libraries, closed its doors in March 2020 and did not reopen until August 2020. Upon reopening, it employed reduced hours of operation, gradually increasing over the following three years. It was only in Spring 2022 that hours returned to similar levels to those offered in 2019. By that time, the service model had changed to a study hall model, with no staffed service desk on Sunday-Thursday between 10:00 p.m. and 12:00 a.m. or on Friday and Saturday evenings due to low circulation numbers and low reported staff interactions with users. To date, users have not formally reported dissatisfaction with this study hall model for extended hours. Additionally, the library looked at gate count data by hour and removed two hours on Sunday (8:00 a.m.–10:00 a.m.) due to lower entrances during that time. Specific student demand for study space played a key role in this change. The pandemic impacted physical library use and further analysis on post-pandemic usage trends is warranted, both within Laupus Library and across health sciences libraries, such as an examination of overarching impacts on gate counts between 2020 and the present, use of librarian services during the hours when librarians are present, and the ways that libraries adjusted operational models throughout the pandemic.

## CONCLUSION

The change in temporal patterns of library use during extended hours justified the change in operational hours and demonstrated the need for extended hours, without increasing operational costs. Although the COVID-19 pandemic halted the extended hours, the data informed the library’s slow readoption of extended hours following the fall of 2021. While many things have changed in the last several years, student demand for physical library access remains strong.

## LIMITATIONS

Gate counts have known limitations for understanding how a library is being used, along with the potential for counting inaccuracies [45]. Because this study used post-hoc, pre-existing data, head counts were not available to the authors; this limits the authors’ ability to assess which areas of the library were being used and the types of activities in which library users were engaged. Two data points, room reservation data and circulation data, may not accurately reflect rates of library use. For example, anatomical models are frequently checked out by one student for use in group study sessions. The number of students using models may not be accurately reflected in the circulation data. Similarly, room reservations may be inaccurate; many students used unoccupied study rooms without a room reservation and users neither had to verify their arrival on time (or at all) for their reserved study room time, nor could they ‘check out’ from the room. This study could therefore only assess the hours of intended use determined by room reservations. This may have led to either an undercount or an overcount in both study room sessions and hours.

The authors did not assess reference desk statistics due to known limitations of the data. Notably, there is both an issue with the fidelity to entry at time of service event, both broadly as well as in individual variability in recording the reference interaction at all. Additionally, librarians in Laupus Library note their use of Saturdays to complete backlogged work received during the standard business week, which would falsely inflate statistics about librarian services on Saturdays when they worked.

Most notably, this study evaluated outcomes of operational changes, rather than elucidating the reasons students accessed the library during extended hours or the value they put on the extended hours. These questions merit further study.

## Data Availability

Data associated with this article are available in Open Science Framework at https://osf.io/6utk7/.

## References

[1] Laaker S. Keeping the doors open: Exploring 24-hour library access at Washington University in St. Louis. Research Library Issues: A Bimonthly Report from ARL, CNI, and SPARC. 2011 Dec;277:15–25. DOI: 10.29242/rli.277.3.

[2] Ravenwood C, Walton G, Stephens D. Complexity in decision making: Determining university library opening hours. Journal of Librarianship and Information Science. 2019 Jun;51(2):488–96. DOI: 10.1177/0961000617726127.

[3] Sanders M, Hodges C. An overnight success?: Usage patterns and demographics of academic library patrons during the overnight period from 11 pm–8 am. Journal of Access Services. 2014 Oct;11(4):309–20. DOI: 10.1080/15367967.2014.945121.

[4] Bowman AC. 24-hour academic libraries: Adjusting to change. Journal of Access Services. 2013 Oct;10(4):217–39. DOI: 10.1080/15367967.2013.842342.

[5] Sewell BB. 24-hour access: Responding to students’ need for late library hours at the University of Denver. Journal of Access Services. 2013 Jan;10(1):14–27. DOI: 10.1080/15367967.2013.738390.

[6] Wolff C, Schonfeld R. Ithaka S+R US Library Survey 2016. [Internet]. 2017 [cited 28 February 2024]. DOI: 10.18665/sr.303066.

[7] Frederick J, Wolff-Eisenberg C. Ithaka S+R US Library Survey 2019. [Internet]. 2020 [cited 28 February 2024]. DOI: 10.18665/sr.312977.

[8] Hulbert I. US Library Survey 2022: Navigating the New Normal. [Internet]. 2023 [cited 28 February 2024]. DOI: 10.18665/sr.318642.

[9] Cook C. Heath F.M., & Thompson B. “Zones of Tolerance” in Perceptions of Library Service Quality: A LibQUAL+ Study. portal: Libraries and the Academy. 2003 Jan;3(1):113–123. DOI: 10.1353/pla.2003.0003.

[10] Arant W, Benefiel CR. Hours of operation and service in academic libraries: Toward a national standard. Public Services Quarterly. 2002 Jan;1(1):71–85. DOI: 10.1300/j295v01n01_08.

[11] De Santis M, Houghton V, Fontenelle C. “If the library genie granted you three wishes, what would they be?”: Results and lessons learned from an annual user feedback campaign. Med Ref Serv Q. 2017 Jan;36(1):9–18. DOI: 10.1080/02763869.2017.1259886. 28112639

[12] Migdalski A, Moreau E. A matter of timing: Analyzing and adjusting library hours to suit students. Journal of Access Services. 2021 Apr;18(2):91–100. DOI: 10.1080/15367967.2021.1911660.

[13] Dimarco S, Dam SV. Late night in an academic library: Issues, concerns, planning. Library & Archival Security. 1998 Aug;14(2):7–23. DOI: 10.1300/J114v14n02_03.

[14] Richards M. 24/7 Library hours at an urban commuter college. Urban Library Journal. 2016 Mar;22(1):2. <https://academicworks.cuny.edu/ulj/vol22/iss1/2>.

[15] Withers R. Perpetual motion: Running a 24/7 library in a 9 to 5 World [Internet]. 2015 [cited 1 Aug 2023]. <https://sc.lib.miamioh.edu/handle/2374.MIA/5253>.

[16] Fajardo FJ, Roth RP, Dolinsky L. What medical students want: A library survey of the first ten classes of a new college of medicine. Med Ref Serv Q. 2021 Jul;40(3):249–60. DOI: 10.1080/02763869.2021.1945855. 34495801

[17] Ugolini D, Faré C. Twenty-four-hour access to a library collection. Bull Med Libr Assoc. 1991 Jul;79(3):323. <https://www.ncbi.nlm.nih.gov/pmc/articles/PMC225561/>. 1884088 PMC225561

[18] Byrd GD, Shedlock J. The Association of Academic Health Sciences Libraries annual statistics: an exploratory twenty-five-year trend analysis. J Med Libr Assoc. 2003 Apr;91(2):186–202. <https://www.ncbi.nlm.nih.gov/pmc/articles/PMC153160/>. 12883578 PMC153160

[19] Obrig K, Harris CR, Lyons K, Powell S. Implementation of 24/7 library hours provides stronger support to the library’s mission [Internet]. Poster presented at: Mid-Atlantic Chapter of the Medical Library Association Meeting; Oct 2010 [cited 1 Aug 2023]. <https://hsrc.himmelfarb.gwu.edu/libfacpres/36/>.

[20] Kindon R. After-hours access at the hsl [Internet]. Presented at: 2018 Meeting of Upstate New York Science Librarians; Nov 2, 2018; Syracuse University, Syracuse, NY. <https://surface.syr.edu/nyscilib/86/>.

[21] Ogawa R. S. The Landscapes of Health Sciences Libraries. In Crum J, Nuñez A. Essential Leadership Skills for Health Sciences Information Professionals. Medical Library Association Book Series. Rowman & Littlefield Publishers; 2023. p. 3–14. https://escholarship.org/uc/item/08x22380>.

[22] Cisney L. B., Hoover B, Thormodson K. The Technology, Budget, and Other Challenges of Growing Health Systems on Academic Health Sciences Libraries: A Deeper Dive. Journal of Electronic Resources in Medical Libraries, 2022 Aug;19(3), 59–84. DOI: 10.1080/15424065.2022.2113349.

[23] Eldermire ERB. When do veterinary medical students study? Understanding student study habits to inform library operating hours. Issues in Science and Technology Librarianship. 2018 Oct;(90). DOI: 10.29173/istl1746.

[24] Kyrillidou M, Bland L. ARL Academic Health Sciences Library Statistics, 2007-2008. [Internet]. 2009 [cited 21 May 2024]. <https://eric.ed.gov/?id=ED507420>.

[25] Bayley L, Ferrell S, Mckinnell J. Practicing What We Preach: A case Study on the Application of Evidence-Based Practice to Inform Decision Making for Public Services Staffing in an Academic Health Sciences Library. New Review of Academic Librarianship. 2009 OCT;15(2), 235–252. DOI: 10.1080/13614530903245311.

[26] Sayed EN, Laws S, Uthman B. Using time-driven activitybased costing to implement change. Med Ref Serv Q. 2017 Jul;36(3):253–65. DOI: 10.29173/istl1746. 28714814

[27] Scarletto EA, Burhanna KJ, Richardson E. Wide awake at 4AM: A study of late night user behavior, perceptions and performance at an academic library. Journal of Academic Librarianship. 2013 Sep;39(5): 371–377. DOI: 10.1016/j.acalib.2013.02.006.

[28] Ferria A, Gallagher BT, Izenstark A, Larsen P, LeMeur K, McCarthy CA, Mongeau D. What are they doing anyway?: Library as place and student use of a university library. EBLIP. 2017 Mar;12(1):18–33. DOI: 10.18438/B83D0T.

[29] Lawrence P, Weber L. Midnight-2.00 a.m.: What goes on at the library? New Library World. 2012 Nov;113(11/12):528–48. DOI: 10.1108/03074801211282911.

[30] Aronoff N. Surveying medical students to gauge library use and plan for a new medical library. Med Ref Serv Q. 2016 Apr;35(2):187–203. DOI: 10.1080/02763869.2016.1152144. 27054535

[31] Saragossi J, Stevens GA, Scheinfeld L, Koos JA. Leveraging Survey Results in Support of a Library Renovation: A Case Study. Medical Reference Services Quarterly. 2020;39(3):238–253. DOI: 10.1080/02763869.2020.1774254. 34000222

[32] Phillips J. Determining gate count reliability in a library setting. EBLIP. 2016 Sep 26;11(3):68–74. DOI: 10.18438/B8R90P.

[33] Martell C. The absent user: Physical use of academic library collections and services continues to decline 1995–2006. The Journal of Academic Librarianship. 2008 Sep;34(5):400–7. DOI: 10.1016/j.acalib.2008.06.003.

[34] Martell C. The elusive user: Changing use patterns in academic libraries 1995 to 2004. College & Research Libraries. 2007 Sep;68(5):435-45. DOI: 10.5860/crl.68.5.435.

[35] Regazzi JJ. Constrained? An analysis of US academic library shifts in spending, staffing, and utilization, 1998–2008. College & Research Libraries. 2012 Sep;73(5):449–68. DOI: 10.5860/crl-260.

[36] Dubnjakovic A. Electronic resource expenditure and the decline in reference transaction statistics in academic libraries. The Journal of academic librarianship. 2012 Mar;38(2):94–100. DOI: 10.1016/j.acalib.2012.01.001.

[37] Freeman GT. The library as place: Changes in learning patterns, collections, technology, and use. In Freeman GT, Bennett S, Demas S, Frischer B, Peterson CA, Oliver KB. Library as place: Rethinking roles, rethinking space. CLIR Publication No. 129. Washington, DC: Council on Library and Information Resources; 2005. p. 1–9. <https://www.clir.org/pubs/reports/pub129/>.

[38] Elmborg JK. Libraries as the spaces between us: Recognizing and valuing the third space. Reference & User Services Quarterly. 2011 Jun;50(4):338–50. DOI: 10.5860/rusq.50n4.338.

[39] Peters T. The library as a third place [Internet]. News & Views from the CMU Libraries; 2019 [cited 1 Aug 2023]. <https://blogs.cmich.edu/library/2019/11/04/the-library-as-a-third-place/>.

[40] Finlay AK, Ram N, Maggs JL, Caldwell LL. Leisure activities, the social weekend, and alcohol use: Evidence from a daily study of first-year college students. J Stud Alcohol Drugs. 2012 Mar;73(2):250–9. DOI: 10.15288/jsad.2012.73.250. 22333332 PMC3281983

[41] Ragsdale JM, Beehr TA, Grebner S, Han K. An integrated model of weekday stress and weekend recovery of students. Int J Stress Manag. 2011 May;18(2):153–180. DOI: 10.1037/a0023190.

[42] Sewell K. An analysis of the effect of Saturday home football games on physical use of university libraries. EBLIP. 2021 Dec;16(4):84–99. DOI: 10.18438/eblip29942.

[43] Missingham R. Libraries and economic value: a review of recent studies. Performance Measurement Metric. 2005 Dec;6(3):142–58. DOI: 10.1108/14678040510636711.

[44] Shepherd J, Vardy K, Wilson A. Quantifying patron timeuse of a public library. Library Management. 2015 Aug;36(6/7):448–61. DOI: 10.1108/LM-09-2014-0110.

[45] Breakenridge S. Rowan University Libraries’ Head-Counting Study. [Internet]. 2018 [cited 28 February 2024]. https://rdw.rowan.edu/lib_scholarship/9/.

