# Supplementary material for: Shifting temporal patterns in physical usage of a health sciences library following the extension of operational hours
Source: J Med Libr Assoc. 2024 Oct 7;112(4):364–71. doi: 10.5195/jmla.2024.1812 (PMC11486077; doi:10.5195/jmla.2024.1812)
Supplement: Supplementary file 1 — Appendix A: Supplementary Tables [file jmla-112-4-364-s01.docx]

Supplementary Table 1. Changes in Gate Counts Pre- and Post-Implementation of Extended Hours

|  | Friday | | Saturday | | Sunday | |
| --- | --- | --- | --- | --- | --- | --- |
|  | Pre-Extended Hours | Post-Extended Hours | Pre-Extended Hours | Post-Extended Hours | Pre-Extended Hours | Post-Extended Hours |
| Total Gate Count Entries | 10758 | 11515 | 5424 | 7184 | 8789 | 10397 |
| Gate Counts Entries during Extended Hours of Operations |  | 964 |  | 790 |  | 2155 |

Supplementary Table 2. Percentage of Room Reservation Hours Occurring During Extended Hours of Operation

|  | Room Reservation Hours During Extended Hours of Operation | Room Reservation Hours During Regular Hours of Operation* | % Room Reservation Hours Occurring During Extended Hours of Operation |
| --- | --- | --- | --- |
| Spring | 1185.5 | 5502 | 17.73% |
| Summer | 193.5 | 1022.5 | 15.91% |
| Fall | 1241 | 5916.5 | 17.34% |

* Regular Hours of Operation here are the hours of day during which Laupus had been open on Fridays, Saturdays, and Sundays for the pre-extended hours period.

Supplementary Figure 1. Changes in Study Room Use by Hour of the Day, Pre- and Post-Implementation of Extended Hours, by Weekend Day and Semester


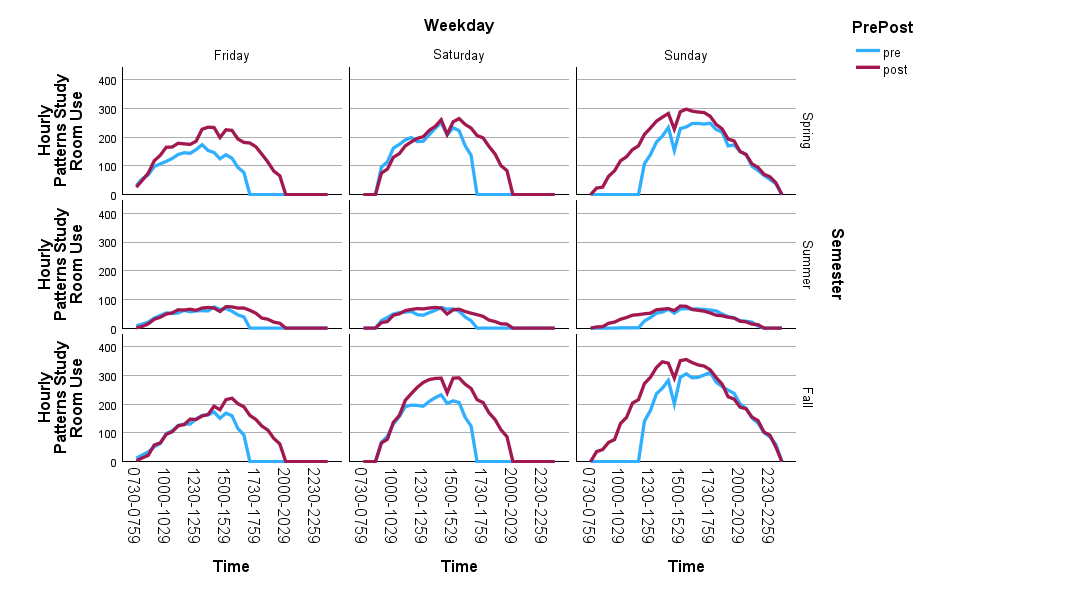


Supplementary Figure 2. Changes in Study Room Reservation Start Time, Pre- and Post-Implementation of Extended Hours, by Weekend Day and Semester


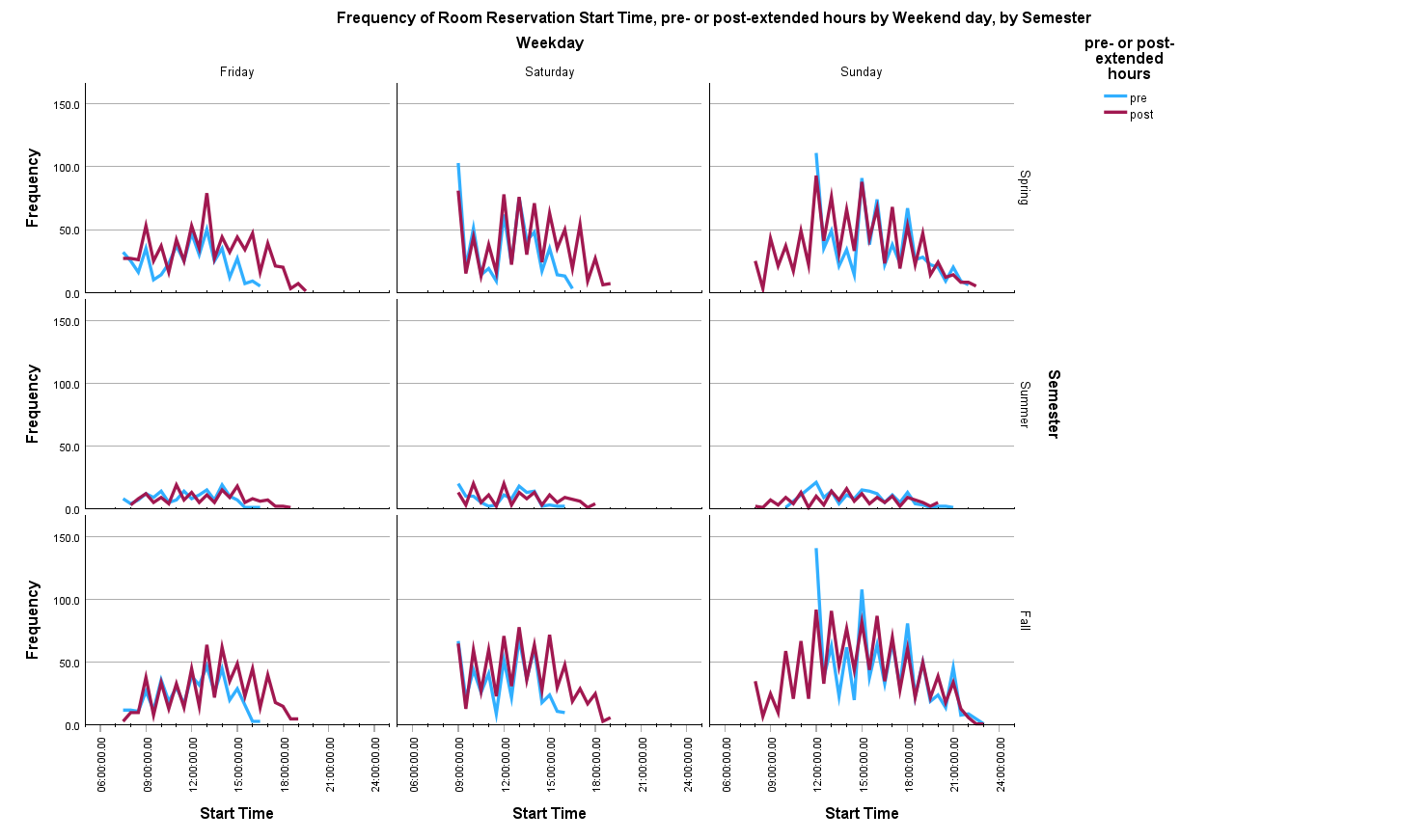


Supplementary Figure 3. Changes in Study Room Reservation End Time, Pre- and Post-Implementation of Extended Hours, by Weekend Day and Semester


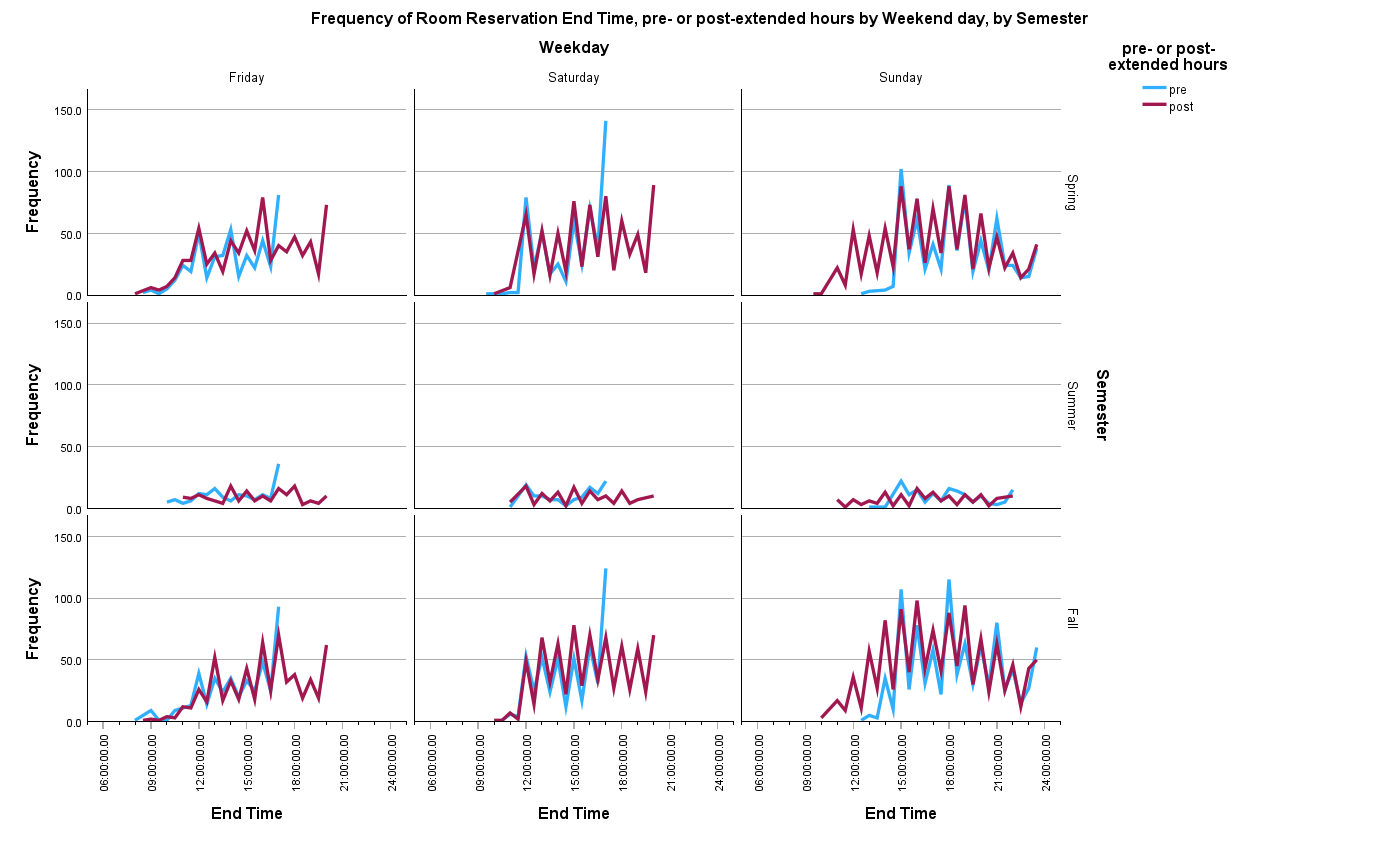


Supplementary Figure 4. Changes in Circulation by Item type for each weekend day, pre- and post-implementation of extended hours


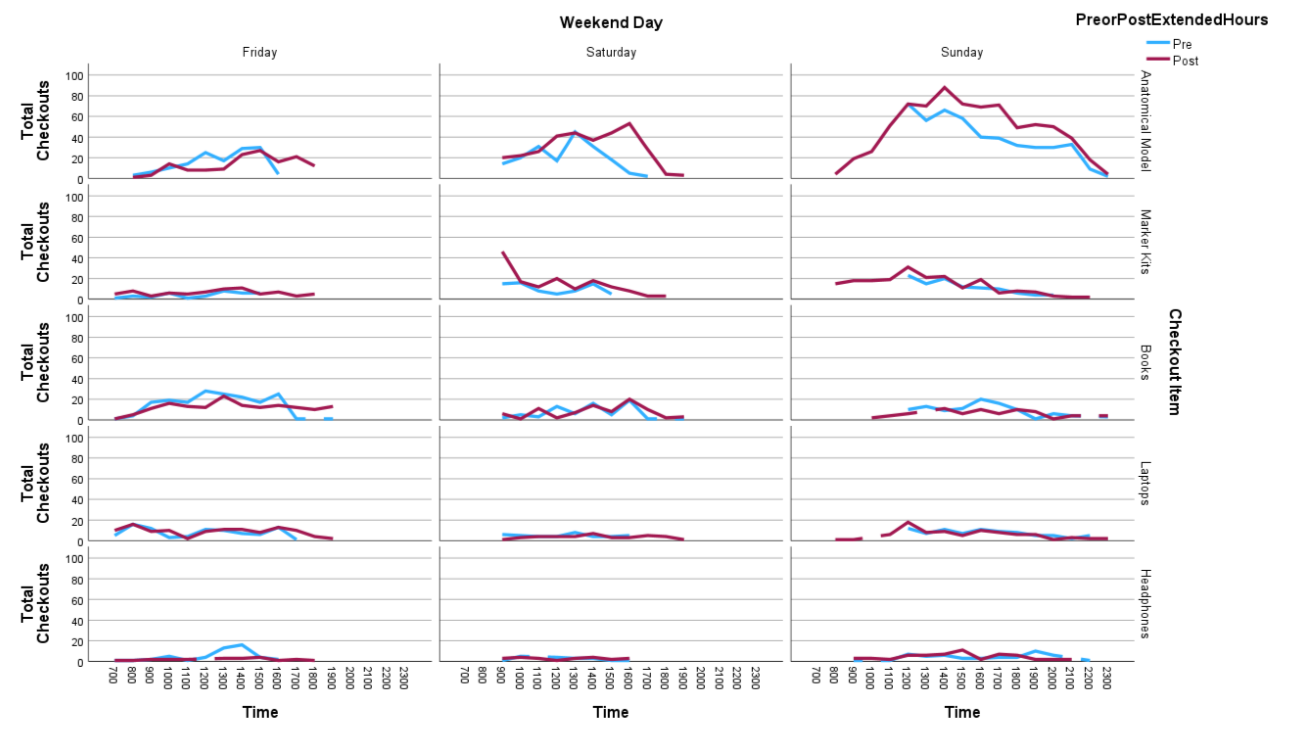

* Checkout numbers for all other item types available for circulation were too small to be included here. The five items listed here account for between 83-96% of all checkouts for Fridays, Saturdays, and Sundays during the study period.
